# Impact of Different Strategies for Delivering Supplemental Zinc on Selected Fecal Markers of Environmental Enteric Dysfunction among Young Laotian Children: A Randomized Controlled Trial

**DOI:** 10.4269/ajtmh.20-0106

**Published:** 2020-07-06

**Authors:** Guy-Marino Hinnouho, K. Ryan Wessells, Maxwell A. Barffour, Somphou Sayasone, Charles D. Arnold, Sengchanh Kounnavong, Sonja Y. Hess

**Affiliations:** 1 Department of Nutrition, Institute for Global Nutrition, University of California, Davis, Davis, California;; 2 Helen Keller International, Washington, District of Columbia;; 3 Public Health Program, College of Health and Human Services, Missouri State University, Springfield, Missouri;; 4 Lao Tropical and Public Health Institute, Vientiane, Lao People’s Democratic Republic

## Abstract

The objective of this study was to assess the impact of different strategies for delivering supplemental zinc on fecal myeloperoxidase (MPO), neopterin (NEO), and calprotectin (CAL) among young Laotian children. In a double-blind controlled trial, children aged 6–23 months were randomized to receive either daily preventive zinc (PZ) tablets (7 mg/day), daily micronutrient powder (MNP; containing 10 mg zinc and 14 other micronutrients), therapeutic zinc (TZ) supplements for diarrhea treatment (20 mg/day for 10 days), or daily placebo powder and followed for ∼36 weeks. Stool samples were collected at baseline and endline. Fecal MPO, NEO, and CAL concentrations were determined in a randomly selected subsample of 720 children using commercially available ELISA kits. At baseline, the mean age was 14.1 ± 4.9 months and prevalence of stunting was 39%. The endline prevalence of stunting was 43%; there was no overall treatment effect on physical growth in the parent trial. At endline, the mean (95% CI) MPO in the PZ group was 1,590 [1,396; 1,811] ng/mL and did not differ from that in the MNP (1,633 [1,434; 1,859] ng/mL), TZ (1,749 [1,535; 1,992] ng/mL), and control (1,612 [1,415; 1,836] ng/mL) groups (*P* = 0.749). Similarly, there was no overall treatment effect on NEO and CAL concentrations (*P* = 0.226 and 0.229, respectively). In this population, the provision of PZ or TZ supplements or MNP had no impact on growth or environmental enteric dysfunction (EED) as assessed by fecal MPO, NEO, and CAL. Additional research is needed to better understand the etiology and proposed mechanisms of EED pathogenesis.

## INTRODUCTION

Linear growth faltering (stunting) is a major public health concern affecting millions of children younger than 5 years in low- and middle-income countries (LMICs).^1^ Stunting is difficult to reverse beyond the first 1,000 days after conception and has lifelong consequences on health and development.^2^ Its pathogenesis is poorly understood and is postulated to be multifactorial, likely involving inadequate intrauterine and postnatal nutrition, recurrent infections, and poor environmental conditions.^3–5^ In the past years, nutrition-specific and infection control interventions aimed at reducing stunting have yielded moderate to no benefits on growth faltering.^6,7^

Environmental enteric dysfunction (EED), originally termed tropical enteropathy,^8^ is an acquired subclinical inflammation of the small bowel mucosa characterized by villous atrophy, altered barrier integrity, and enteric immune cell proliferation, leading to reduced nutrient absorption and increased intestinal permeability.^9–13^ The specific cause of EED remains unknown, but it has been postulated that EED develops as the result of chronic exposure to enteropathogens caused by regular fecal–oral contamination.^14–17^ Environmental enteric dysfunction is reported to be endemic among infants and children living in LMICs. Thus, it may play a potential role in linear growth faltering,^18,19^ and its severity has been found to be inversely associated with linear growth.^20–23^

Intestinal biopsy via endoscopy is considered the gold standard for the assessment of EED.^24^ However, this procedure is invasive, expensive, requires a high level of expertise, and only measures pathological changes. Over the years, EED has been assessed using a wide range of noninvasive biomarkers measured in urine, blood, or stool. These biomarkers have been investigated and tested for their hypothesized functions of intestinal absorption and mucosal permeability, enterocyte mass and function, intestinal and systemic inflammation, microbial translocation, and immune activation.^4,10,25^ Fecal markers have emerged as a new means of characterizing EED and classifying its severity and include myeloperoxidase (MPO), neopterin (NEO), and calprotectin (CAL). Myeloperoxidase is a specific enzyme of neutrophil activity in the intestinal mucosa^26^; NEO is produced by macrophages or dendritic cells on stimulation by interferon gamma released during pro-inflammatory responses by Th1 lymphocytes^27^; and CAL is a calcium- and zinc-binding protein that inhibits metalloproteinase and is the major protein found in monocytes and macrophages.^28^ To the best of our knowledge, these fecal biomarkers of EED have not been compared with endoscopic findings in young children because intestinal biopsy is technically and ethically not feasible in young children. These biomarkers do not correspond to an increased intestinal permeability but instead are indicative of intestinal inflammation, and NEO is also a biomarker of microbial translocation and immune activation.^9,19^ It is important to note that there are no specific cutoffs for these biomarkers.

Zinc is involved in numerous metabolic processes as a catalyst, a regulatory ion, or structural element of proteins,^29^ and deficiency in young children has been associated with both linear growth failure^1^ and EED.^30^ In infants and young children in LMICs, preventive zinc (PZ) supplementation has been shown to increase linear and ponderal growth and reduce the incidence of diarrhea.^31,32^ In addition, the WHO has recommended therapeutic zinc (TZ) supplementation along with oral rehydration therapy during episodes of diarrhea, and this strategy has been shown to reduce the duration and severity of the disease.^33,34^ These beneficial impacts of PZ and TZ supplementation on growth and diarrhea are postulated to be partially mediated through an increase in villous height and intestinal absorptive capacity.^35^

It is well established that children who are deficient in one micronutrient are often at risk for other deficiencies. Thus, supplementation with multiple micronutrients such as micronutrient powders (MNPs) is a preferred approach to improve young children’s nutrition and health. Worldwide, a considerable number of MNP intervention studies have been conducted in different settings, and their efficacy in the prevention of iron deficiency and anemia has been demonstrated.^36,37^ However, the beneficial impact observed with PZ supplementation on growth and diarrhea has not been demonstrated with MNPs. Moreover, a recent meta-analysis of MNPs found an increase in diarrhea incidence in children receiving MNPs compared with controls,^37^ which was assumed to be due to potentially adverse effects of iron. Only a few randomized controlled trials have assessed the impact of zinc or MNP supplementation on indicators of EED, and results from these studies were inconsistent. In these studies, EED has been measured either by the lactulose-to-mannitol (L:M) ratio, a dual sugar absorption test,^35,38,39^ CAL,^40,41^ or confocal laser endomicroscopy and mechanistic target of rapamycin complex 1 nutrient responsiveness.^42^

In light of the previously described lack of a beneficial impact of standard MNP formulations on zinc-related functional outcomes and concerns of potential adverse effects of MNPs, we used a new MNP formulation containing a higher amount of zinc (10 mg) and a lower amount of iron (6 mg).^43^ We hypothesized that zinc supplementation, provided alone or as part of the high-zinc low-iron–containing MNP, will improve EED as measured by fecal MPO, NEO, and CAL. Thus, our primary objective was to assess the impact of different strategies for delivering supplemental zinc on MPO, NEO, and CAL. As secondary objectives, we first assessed whether baseline MPO, CAL, and NEO modified the intervention impact on growth outcomes; second, we explored associations between baseline MPO, CAL, and NEO and subsequent linear growth; and third, we examined baseline and endline associations between these EED biomarkers and concurrent growth indicators.

## MATERIALS AND METHODS

### Study design and population.

The Lao Zinc Study was a community-based randomized, double-blind, placebo-controlled trial, implemented from September 2015 until April 2017 in rural communities in Khammouane Province, central Lao People’s Democratic Republic (Lao PDR). The study protocol and the consent procedure were approved by the National Ethics Committee for Health Research, Ministry of Health, Lao PDR, and the Institutional Review Board of the University of California, Davis (UC Davis). This trial is registered at https://clinicaltrials.gov (NCT02428647).

The primary objective of the Lao Zinc Study was to determine the impact of two forms of daily preventive supplementation (zinc tablets and MNP) versus TZ supplementation for diarrhea on young children’s physical growth and other health outcomes. Details of the study protocol have previously been published elsewhere.^43^ Briefly, children were considered eligible to participate if they were aged 6–23 months, their families accepted weekly home visits, planned residency within the catchment area for the duration of the study, and one of the child’s primary caregivers (mother, father, or legal guardian) provided a written informed consent (documented by either a signature or a fingerprint in the presence of a neutral witness). Children were ineligible to participate if one of the following criteria was present: severe anemia (Hb < 70 g/L), weight-for-length *z*-score (WLZ) < −3SD with respect to WHO 2006 growth standards,^44^ presence of bipedal edema, severe illness warranting hospital referral, congenital abnormalities potentially interfering with growth, chronic medical condition (e.g., malignancy) requiring frequent medical attention, known HIV infection of the index child or the child’s mother, current consumption of zinc supplements, or current participation in another clinical trial.

### Randomization and intervention products.

For the parent trial, a total of 3,433 children were enrolled and randomized into one of four intervention groups using a block randomization scheme, with block lengths of 4 or 8, generated by a UC Davis statistician. If multiple eligible siblings resided in the same household, only the youngest was enrolled. In the case of twins, both twins were enrolled and assigned to the same group, but only one was selected randomly for inclusion in the data analyses.

Eligible children were individually randomized either to 1) the PZ group, receiving 7 mg of a daily preventive dispersible zinc supplement plus a placebo therapeutic tablet for diarrhea; or 2) the MNP group, receiving a daily preventive MNP containing 10 mg zinc and 14 other micronutrients plus a placebo therapeutic tablet for diarrhea; or 3) the TZ group, receiving a daily preventive placebo tablet plus 20 mg of TZ for diarrhea for 10 days; or 4) the control group, receiving a daily placebo powder plus a therapeutic placebo tablet for diarrhea. As mentioned earlier, the tested MNP formulation contained a higher amount of zinc (10 mg zinc as zinc gluconate) and a lower amount of iron (6 mg iron as ferrous fumarate) than standard MNP formulations.^43^ In addition, the MNP contained 0.56 mg copper as copper sulfate anhydrous, 17 μg selenium as selenium selenite, 90 μg iodine as potassium iodate, 400 μg RE vitamin A, 5 μg vitamin D (cholecalciferol), 5 mg vitamin E (dl-α-tocopherol acetate), 30 mg ascorbic acid, 0.5 mg thiamine, 0.5 mg riboflavin, 6 mg niacin, 0.5 mg vitamin B-6, 0.9 μg vitamin B-12, and 150 μg folic acid. All children received oral rehydration salts (ORS) to be taken during diarrhea episodes. Oral rehydration salts were part of the diarrhea treatment kit, which was given during enrollment, with instructions to store it in the home and use it for the treatment of a diarrhea episode in the study child. As previously described, caregiver-reported adherence to the preventive supplements was high and resulted in a daily supplemental zinc intake of ∼6.5 mg for the PZ and ∼9.0 mg for the MNP group over the duration of the study.^45^ Children in the TZ group consumed an average of seven of 10 prescribed tablets per diarrhea episode, which resulted in an equivalent of ∼0.8 mg zinc/day over the course of the study.^46^

The PZ, TZ, and placebo tablets were produced by Nutriset SAS (Malaunay, France). The powder supplements (MNP and placebo) were produced by DSM Fortitech Asia Pacific Sdn Bhd (Banting, Malaysia). Caregivers were instructed to dissolve the tablet supplements (one dose per day for PZ and one tablet daily for 10 days as part of diarrhea management for TZ) with clean water or breast milk and spoon feed the child 30 minutes before or after a meal. For the powder supplements, they were advised to mix the entire content of the sachet with a semisolid or mashed food.

### Data collection.

At enrollment, children’s weight, length, and mid-upper arm circumference (MUAC) were measured in duplicate by trained anthropometric teams, using standardized procedures.^47^ Unclothed or lightly dressed children were weighed to the nearest 20 g (SECA 383 balance, Hamburg, Germany). Children’s recumbent length (SECA 416 length board, Hamburg, Germany) and MUAC (left arm; Tri-Colored Single-Slotted Insertion Tape, Weigh and Measure, Olney, MD) were measured to the nearest 0.1 cm. If the duplicate measurements differed by > 0.1 kg for weight or by > 0.5 cm for recumbent length and MUAC, the measurement was repeated a third time, and the average of the two measurements with the lowest absolute difference was calculated. Anthropometric measurements were repeated at midpoint (after ∼18 weeks) and at endline (∼36 weeks). Maternal weight (SECA 874, Hamburg, Germany) and height (SECA 213, Hamburg, Germany) were measured once. A total of four standardization sessions were implemented over the course of the study to compare the performance of anthropometry teams among themselves and with their supervisors^48^; results of these standardizations have been reported elsewhere.^49,50^ Hemoglobin concentration was determined in a capillary blood sample (HemoCue^®^ Hb301, HemoCue AB, Angelholm, Sweden), and anemia status was assessed at baseline and endline.

Information on maternal and household demographic and socioeconomic status (e.g., education, occupation, ethnicity, household size and composition, housing material, household assets, and land ownership), food security, and hygiene and sanitation practices of eligible children was obtained at baseline. Information on infant and young child feeding practices (breastfeeding, formula feeding, and 24-hour and 7-day food frequency questionnaire) was collected at baseline and every 4 weeks during the intervention period.

Children enrolled in the trial remained under observation and received their assigned supplements daily for a period of ∼36 weeks. Each household was visited weekly by a morbidity surveillance worker who recorded reported morbidity symptoms for each day of the previous week and delivered the respective preventive supplements. Recorded morbidity symptoms included fever, diarrhea (number and consistency of stools), respiratory symptoms (cough and nasal discharge), and any other symptoms of concern. Axillary temperature was measured once every 4 weeks and whenever fever was reported within 24 hours of the home visit.

### Stool sample collection and EED biomarker analyses.

**Figure 1.**
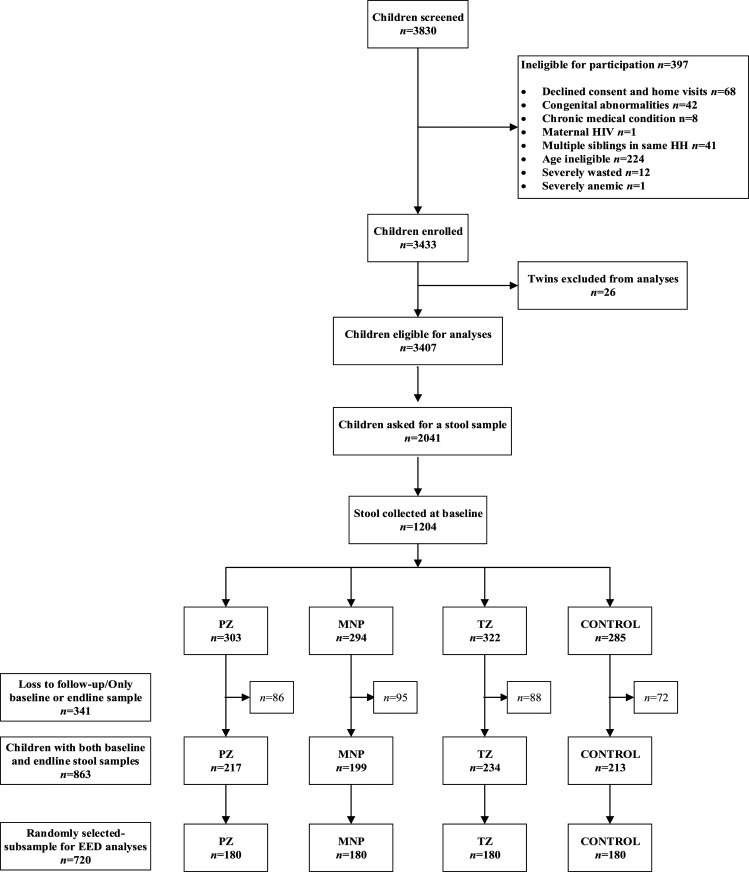
Lao Zinc Study flow diagram for stool sample collection.

Because of the timing and allocation of additional research funding for the present sub-study and given initial difficulties in obtaining stool samples from young children, stool collection was started mid-study and was attempted in approximately 2000 children (*n* = 2,041, Figure 1). Stool sample collection was attempted on two consecutive days at both baseline (before the initiation of the supplementation) and endline. Samples were collected on consecutive days at each time point to minimize failure to detect low-intensity helminth infection. Disposable diapers were distributed to caregivers with the instruction to place a diaper on the child immediately before sleep in the evenings before the days of stool collection. Caregivers were instructed to return to the study site the following day with all diapers containing any stool. At baseline, the 2-day collection requirement necessitated that caregivers be given diapers during the community sensitization session, 1 day before the study enrollment. Thus, oral consent specific for the stool collection was obtained during the community sensitization session, and stool samples were only stored and analyzed for eligible children with written parental informed consent. Among children presenting with diarrhea at enrollment (*n* = 34), stool collection was not repeated on a second day because preventive and therapeutic supplementation were initiated immediately following diagnosis. These children only provided one stool sample and were included in the random selection for the present study, if they also provided stool samples at endline. Stool collection was attempted for all children on two consecutive days at endline.

A mobile field laboratory was set up for immediate stool sample processing. The approximate time of defecation was recorded based on caregiver report, and in cases where more than one diaper was provided, stool samples from the most recent defecation were aliquoted into a container by trained laboratory technicians. Samples from that container were further aliquoted into 1.5 mL amber microcentrifuge tubes and immediately stored at −18°C in a portable freezer (CF-025, Dometic, Solna, Sweden). All samples were subsequently stored at −20°C for up to 7 days, after which some aliquots were transferred to a −80°C freezer,^43^ as specified by ELISA protocols.

Stool samples were shipped on dry ice to the University of California, Davis, USA, where the MPO aliquots were stored at −80°C and the NEO and CAL aliquots at −20°C. In cases where stool samples were collected at both days of stool collection, the one with the shorter duration since defecation was selected. Samples were then analyzed using commercially available ELISA kits following the manufacturer’s instructions. A stool application system (ALPCO, Salem, NH) was used to dilute the stool samples in the respective buffer of each marker. Fecal MPO was determined after a dilution of 1:500 (ALPCO). Fecal NEO concentrations were measured after an initial dilution of 1:1,300 (GenWay Biotech, San Diego, CA), and CAL was assayed after an initial dilution of 1:2,500 (ALPCO). Absorption or optical density was determined with an ELISA reader (Synergy H1, BioTek Instruments, Winooski, VT) at the wavelength 450 nm against 620 nm as a reference. Extracts from samples out of range of the standard curve were diluted a second time in their respective dilution buffer, and assays were rerun at lower concentrations.

### Sample size for the EED analyses.

A sample size of 179 children (rounded up to 180) per study intervention group was estimated to be able to detect a difference in the mean of any given biomarker of EED between any two intervention groups with an effect size of 0.35, a power of 80%, and a type I error of 5%.^51^ An attrition rate of 20% was considered to account for drop out and possible failure to successfully collect stool samples at both time points (total sample size = 900). Thus, from children who provided stool samples at both baseline and endline, 720 (*n* = 180 per intervention group) were randomly selected for the EED analyses. Sample size estimation was done with the use of SAS software (version 9.4; SAS Institute, Inc., Cary, NC).

### Definitions.

*Z*-scores for length-for-age (LAZ), weight-for-age (WAZ), and weight-for-length (WLZ) were calculated according to the WHO 2006 child growth standards.^44^ Stunting, underweight, and wasting were defined as LAZ, WAZ, and WLZ < −2SD, respectively. Low MUAC was defined as MUAC ≤ 12.5 cm.

A child was considered breastfed if breastfeeding was reported at least once in the past month. Information on infant and young children feeding practices (breastfeeding, dietary diversity, and food frequency) was used to estimate the adequate dietary diversity, minimum meal frequency, and consumption of iron-rich foods as specified by the WHO.^52,53^ Food security was defined using the household food insecurity access scale.^54^ Principal component analysis was applied to available indicators of household socioeconomic status, education, income, ownership of lands, and hygiene and sanitation practices to derive a SES index.^55^

### Statistical analyses.

A statistical analysis plan describing the statistical procedures was developed and published before the analyses.^56^ Analyses were done based on complete-case intention-to-treat, and the intervention group was considered the primary exposure variable. All analyses were undertaken using Stata 14 (StataCorp 2015, College Station, TX).

ANCOVA regression models were used to assess treatment effects in minimally adjusted models controlling for baseline measurement of the outcome, age of the child at enrollment, and district of residence at enrollment. A secondary fully adjusted analysis was performed controlling for additional prespecified variables at baseline determined to be associated with outcome (*P* < 0.1) in bivariate models. Baseline and endline concentrations of MPO, NEO, and CAL were log transformed for analysis. Effect modification was tested using prespecified baseline covariates by including an interaction term in the models.

The treatment effect on growth outcomes has previously been reported,^50^ but EED data were unavailable at that time. With the available data, we investigated the potential effect modification of EED (as continuous effect modifiers) at baseline on growth outcomes at midpoint and endline. Environmental enteric dysfunction marker concentrations were then categorized in tertiles, and an interaction term was incorporated in the statistical models and further examined if marginally significant (*P* < 0.1).

The association between baseline MPO, NEO, and CAL and subsequent change in LAZ (baseline to midpoint and baseline to endline) was examined with linear regression controlling for LAZ at baseline, age at enrollment, health district, and intervention group.

Baseline and endline associations between the EED biomarkers and concurrent growth indicators were explored using linear regression models adjusted for age of the child at enrollment, gender, district of enrollment, and intervention group (only for the endline associations).

## RESULTS

### Baseline characteristics of participants.

Of the 3,830 children screened for eligibility, 3,433 were enrolled and 3,407 were individually randomized in the parent trial (Figure 1). Among them, 2,041 children were invited to provide a stool sample, and stool samples were collected from 1,204 children at baseline. Of these, 863 provided stool samples at both baseline and endline, and 720 children with stool samples at both time points (*n* = 180 per intervention group) were then randomly selected for the EED analyses.

At baseline, the mean age was 14.1 ± 4.9 months (Table 1). Approximately 77% of children were breastfed in the previous 4 weeks, and 5% and 47% met the WHO definition of adequate dietary diversity and minimal meal frequency, respectively; 38% of the households reported to be food secure. The prevalence of stunting, underweight, and wasting was 39%, 26%, and 8%, respectively, and 57% of children were anemic at baseline. The median [IQR] concentrations of fecal MPO, NEO, and CAL at baseline were 2,710 [1,249–7,305] ng/mL, 629 [176–1,829] nmol/g, and 133 [47–279] µg/mL, respectively.

**Table 1:** Selected child, maternal, and household characteristics of the study participants at baseline, by intervention group*

Characteristic	All (*n* = 720	Preventive zinc (*n* = 180)	Micronutrient powder (*n* = 180)	Therapeutic zinc (*n* = 180)	Control (*n* = 180)
Age (months)	14.1 ± 4.9	14.1 ± 4.8	14.2 ± 4.8	13.8 ± 5.1	14.3 ± 4.9
Females, *n* (%)	360 (49.5)	86 (47.0)	90 (49.7)	82 (45.6)	102 (55.7)
Breastfeeding†, *n* (%)	435 (76.7)	112 (80.6)	112 (83.0)	115 (76.2)	96 (67.6)
Adequate dietary diversity†‡	29 (5.1)	8 (5.8)	7 (5.2)	6 (4.0)	8 (5.6)
Minimal meal frequency†§	268 (47.3)	68 (48.9)	74 (54.8)	64 (42.4)	62 (43.7)
Consumption of iron-rich foods†	367 (64.6)	95 (68.4)	80 (59.3)	95 (62.5)	97 (68.3)
Child anthropometric measures					
Length (cm)	72.5 ± 5.5	72.4 ± 5.2	72.2 ± 5.3	71.8 ± 5.6	72.6 ± 5.3
Weight (kg)	8.3 ± 1.3	8.3 ± 1.2	8.2 ± 1.3	8.2 ± 1.3	8.3 ± 1.2
Mid-upper arm circumference (cm)	13.7 ± 1.0	13.7 ± 0.9	13.8 ± 1.1	13.7 ± 0.9	13.7 ± 0.9
Length-for-age *z*-score	−1.76 ± 1.01	−1.75 ± 0.94	−1.81 ± 1.06	−1.82 ± 1.11	−1.67 ± 0.89
Weight-for-age *z*-score	−1.45 ± 0.93	−1.41 ± 0.86	−1.48 ± 1.02	−1.50 ± 1.04	−1.41 ± 0.77
Weight-for-length *z*-score	−0.72 ± 0.94	−0.69 ± 0.92	−0.73 ± 1.05	−0.73 ± 0.94	−0.74 ± 0.83
Stunting	286 (39.3)	70 (38.3)	76 (42.2)	76 (42.2)	64 (35.0)
Wasting	61 (8.4)	13 (7.1)	17 (9.4)	17 (9.4)	14 (7.7)
Underweight	185 (25.5)	46 (25.6)	56 (30.9)	46 (25.6)	37 (20.2)
Environmental enteropathy dysfunction markers					
Myeloperoxidase (ng/mL)	2,710.3 [1,249.2–7,305.0]	2,485.6 [1,181.5–6,089.5]	2,968.9 [1,452.4–6,799.4]	3,571.4 [1,502.9–10,117.9]	2,482.9 [1,123.5–7,517.9]
Neopterin (nmol/g)	629.5 [176.3–1,829.4]	573.2 [191.7–1,894.6]	666.1 [193.8–1,811.7]	788.9 [173.9–1,782.6]	468.3 [141.6–1,849.3]
Calprotectin (µg/mL)	133.1 [47.1–279.6]	131.3 [51.4–265.9]	129.1 [35.2–274.0]	153.3 [55.7–340.0]	125.8 [51.5–267.8]
Hemoglobin concentrations (g/L)	107.0 ± 10.6	106.8 ± 11.0	105.9 ± 10.2	106.8 ± 10.8	108.3 ± 10.4
Anemia (Hb < 110 g/L), *n* (%)	415 (57.1)	106 (57.9)	111 (61.3)	99 (55.0)	99 (55.0)
Maternal education, primary or lower	528 (73.4)	130 (71.0)	133 (74.3)	132 (74.2.6)	133 (74.3)
Maternal body mass index (kg/m^2^)	21.5 ± 2.8	21.7 ± 2.7	21.5 ± 2.9	21.4 ± 2.9	21.5 ± 2.7
Household food insecurity access scale, food secure, *n* (%)	277 (38.2)	62 (34.1)	69 (38.3)	69 (38.3)	77 (42.1)

* Values presented as *n* (%), means ± SDs, or medians [IQRs].

† *n* = 568.

‡ Adequate dietary diversity: Proportion of children aged 6–23 months who receive foods from four or more food groups.

§ Minimal meal frequency: Proportion of breastfed and non-breastfed children aged 6–23 months who receive solid, semisolid, or soft foods the minimum number of times or more.

The children included in this analysis were statistically similar to children participating in the main trial, but who were not part of the EED analyses, in terms of age at enrollment, gender, baseline anthropometric indicators, anemia prevalence and maternal age, and body mass index (all *P* > 0.05). However, they were more likely to be breastfed (77% versus 72%, *P* = 0.018), had lower adequate dietary diversity (5% versus 17%, *P* < 0.001), and had mothers with no education (73% versus 69%, *P* = 0.014), but were less likely from severely food insecure households (10% versus 14%, *P* < 0.001). However, in models adjusted for age, gender, and district of residence, breastfeeding, dietary diversity, maternal education, and food security at baseline were not associated with any of the three selected biomarkers of EED.

### Impact of the intervention on selected biomarkers of EED.

At endline, the minimally adjusted geometric mean (95% CI) concentration of MPO did not differ across the four intervention groups (1,590 [1,396; 1,811] for PZ; 1,633 [1,434; 1,859] for MNP; 1,749 [1,535; 1,992] for TZ; and 1,612 [1,415; 1,836] ng/mL for the control group; *P* = 0.749) (Table 2). Similarly, there was no overall impact of the different interventions of delivering supplemental zinc on both endline concentrations of NEO (201 [164; 245] for PZ; 226 [185; 276] for MNP; 186 [153; 228] for TZ; and 246 [201; 300] nmol/g for the control group; *P* = 0.226) and CAL (35 [28; 45] for PZ; 39 [31; 48] for MNP; 38 [30; 48 for TZ; and 49 [39; 61] µg/mL for the control group; *P* = 0.229) in models adjusted for baseline measurement of fecal biomarker, age of the child at enrollment, and district of residence at enrollment. Similar results were found in fully adjusted models (controlling for covariates in minimally adjusted modes and predefined covariates associated with the outcome). Effect modification of this intervention effect by prespecified covariates was generally insignificant or inconsistent (data not shown).

**Table 2:** Effects of daily PZ, MNP, or TZ for diarrhea on MPO, NEO, and CAL concentrations among young Laotian children

	PZ	MNP	TZ	Control	*P*-value
Endline MPO (ng/mL)	1,590.2 (1,396.2; 1,811.2)	1,633.0 (1,434.3; 1,859.3)	1,748.9 (1,535.4; 1,991.9)	1,611.9 (1,415.4; 1,835.8)	0.749
Endline NEO (nmol/g)	200.7 (164.4; 245.0)	226.0 (185.2; 275.9)	186.4 (152.7; 227.6)	245.7 (201.2; 300.1)	0.226
Endline CAL (µg/mL)	35.4 (28.2; 44.5)	38.5 (30.7; 48.4)	37.9 (30.2; 47.7)	48.8 (38.9; 61.3)	0.229

CAL = calprotectin; MNP = micronutrient powder; MPO = myeloperoxidase; NEO = neopterin; PZ = preventive zinc; TZ = therapeutic zinc. Estimates are means (95% CI). ANCOVA regression models adjusted for baseline value of outcome of interest, age at enrollment, and district of residence at enrollment were used to examine the difference in mean MPO, NEO, and CAL at endline. Results shown as geometric mean (95% CI). MPO, NEO, and CAL were log transformed and then the estimates were back-transformed using Microsoft Excel’s (version 8.1) exponential function.

### Effect modification by baseline biomarkers of EED on growth outcomes.

As previously reported elsewhere, there was no overall treatment effect on physical growth in the parent trial.^50^ To explore whether this lack of impact may partially be due to EED, we tested for effect modification by baseline MPO, NEO, and CAL concentrations on the impact of the study interventions on physical growth after ∼18 weeks (midpoint) and ∼36 weeks (endline) (Table 3).

**Table 3:** Effect modification by baseline MPO, NEO, and CAL concentrations on the impact of study intervention on midpoint and endline growth outcomes among young Laotian children

	MPO	NEO	CAL
Midpoint			
Length	0.451	0.290	0.966
Weight	0.953	0.519	0.772
MUAC	0.775	0.171	0.948
Low MUAC (≤ 12.5 cm)	0.867	0.734	0.200
LAZ	0.480	0.231	0.972
Stunting	0.841	0.929	0.358
WAZ	0.824	0.575	0.755
Underweight	0.644	0.300	0.984
WLZ	0.708	0.163	0.507
Wasting	**0.058**	0.602	0.696
Endline			
Length	0.640	0.819	0.768
Weight	0.601	0.841	0.423
MUAC	0.245	0.425	0.415
Low MUAC (≤ 12.5 cm)	**0.056**	0.438	0.483
LAZ	0.630	0.754	0.710
Stunting	0.252	**0.026**	**0.017**
WAZ	0.605	0.835	0.543
Underweight	0.182	0.743	0.769
WLZ	0.894	0.602	**0.074**
Wasting	**0.005**	0.316	0.385

CAL = calprotectin; LAZ = length-for-age *z*-score; MPO = myeloperoxidase; NEO = neopterin; WLZ = weight-for-length *z*-score; MUAC = mid-upper arm circumference. Estimates are *P*-value for interaction. ANCOVA regression models adjusted for baseline value of outcome of interest, age at enrollment, and district of residence at enrollment were used to examine the effect modification. Bold indicates values are statistically significant or marginally significant *P*-values.

Myeloperoxidase modified the effect of the intervention on wasting at midpoint (*P* = 0.058) and on wasting and low MUAC at endline (*P* = 0.005, 0.056). These growth outcomes had low prevalence, and stratified models did not converge. Evaluation of unmodeled outcome prevalences by the treatment group and MPO tertile revealed no effect pattern and was not consistent across time point. After controlling for multiple hypothesis testing, these interactions were not statistically significant.

Baseline NEO and CAL concentrations modified the effect of the intervention on endline stunting (*P* for interaction = 0.026 and 0.017, respectively) such that among children in the lowest tertile of NEO concentrations at baseline, there was a trend toward a higher prevalence of stunting at endline in the MNP (∼51%) and TZ (∼50%) versus PZ (∼36%) and control (∼37%) groups; among children in the middle tertile of NEO concentrations at baseline, the prevalence of endline stunting was similar (∼39–41%) in the different groups; among children in the highest tertile of NEO concentrations at baseline, there was a trend toward a higher prevalence of stunting at endline in the PZ (∼52%) versus MNP (∼45%), TZ (∼44%), and control (∼48%) groups (Supplemental Figure 1). A similar pattern was observed across tertiles of baseline CAL concentrations (Supplemental Figure 2). Moreover, baseline concentration of CAL modified the impact of the interventions on WLZ (*P* for interaction = 0.074) such that among children in the lowest tertile of CAL concentrations at baseline, there was a trend toward a lower WLZ at endline in the PZ (∼−0.82SD) versus control (∼−0.71SD), TZ (∼−0.65SD), and MNP (∼−0.58SD) groups; among children in the middle tertile of CAL concentrations at baseline, WLZ was similar across groups (∼−0.63;−0.69); among children in the highest tertile of CAL concentrations at baseline, there was a trend toward a higher WLZ at endline in the control (∼−0.60) versus MNP (∼−0.70), PZ (∼−0.69), and TZ (∼−0.69) groups (Supplemental Figure 3). However, after multiple hypothesis testing, all the effect modifications were no longer significant.

### Associations between biomarkers of EED at baseline and subsequent linear growth.

Baseline MPO was associated with subsequent acquisition of linear growth failure at 16–20 weeks but not 32–40 weeks after enrollment (change in LAZ −0.029 [−0.054, −0.003]; *P* = 0.027), whereas NEO was marginally associated with subsequent acquisition of linear growth failure 32–40 weeks after enrollment (−0.019 [−0.041, 0.003]; *P* = 0.086) (Table 4). Calprotectin concentration at baseline was not associated with subsequent linear growth at either time point.

**Table 4:** Associations between baseline MPO, NEO, and CAL concentrations and changes in LAZ over time among young Lao children*

Baseline variable	Change in LAZ 16–20 weeks after baseline†	*P*-value	Change in LAZ 32–40 weeks after baseline†	*P*-value
MPO, ng/mL	−0.029 (−0.054, −0.003)	**0.027**	−0.008 (−0.033, 0.017)	0.526
NEO, nmol/g	−0.018 (−0.040, 0.005)	0.118	−0.019 (−0.041, 0.003)	0.086
CAL, µg/mL	−0.001 (−0.021, 0.020)	0.946	−0.003 (−0.024, 0.017)	0.728

* CAL = calprotectin; MPO = myeloperoxidase; NEO = neopterin. MPO, CAL, and NEO concentrations are log transformed. Bold indicates values are statistically significant or marginally significant *P*-values.

† Estimates are regression coefficients and 95% CI and models were adjusted for baseline value of outcome of interest, treatment group, age at enrollment, and district.

### Baseline and endline associations between biomarkers of EED and concurrent growth indicators.

There were no association between baseline MPO and NEO and baseline indicators of growth status. There was a significantly positive association between CAL and MUAC at baseline (regression coefficient [95% CI] = 0.15 [0.02, 0.27]; *P* = 0.021) (Supplemental Table 1); furthermore, wasted children at baseline had higher concentrations of CAL (0.42 [0.02, 0.83]; *P* = 0.042), whereas stunted children had lower concentrations of CAL, but the association was marginal (−0.20 [−0.44, 0.04]; *P* = 0.098).

At endline, there were no observed associations between endline NEO and indicators of growth at endline. However, there was a marginal negative association between MPO and WLZ (−0.07 [−0.16, 0.01]; *P* = 0.093) and between CAL and weight (−0.11 [−0.24, 0.00]; *P* = 0.060) (Supplemental Table 2). Endline CAL was negatively associated with WAZ (−0.14 [−0.28, −0.00]; *P* = 0.044) and WLZ (−0.15 [−0.29, −0.00]; *P* = 0.044) but positively associated with underweight (0.32 [0.04, 0.60]; *P* = 0.024).

## DISCUSSION

### Summary of main findings.

Zinc deficiency has been linked with both EED and linear growth failure, and EED severity has been inversely associated with linear growth. In the present study, we hypothesized that zinc supplementation would improve EED and assessed the impact of different strategies for delivering supplemental zinc on selected fecal markers of EED among young Laotian children. Our results show that daily zinc supplementation provided alone as a single nutrient (7 mg zinc) or as part of a MNP (10 mg zinc) and TZ supplementation given for the treatment of diarrhea (20 mg zinc per day for 10 days) had no impact on concentrations of fecal MPO, NEO, and CAL among 6–23 months aged Laotian children. In addition, MPO, NEO, and CAL concentrations at baseline did not consistently modify the effect of the intervention on midpoint and endline growth indicators. Moreover, after multiple hypothesis testing, all effect modifications were no longer significant. Concentrations of EED markers at baseline were minimally associated with subsequent linear growth failure; only the relationship between MPO and linear growth measured 16–20 weeks after enrollment was significant. In contrast, only CAL concentrations at baseline and endline appeared to be associated with concurrent growth indicators at baseline and endline, respectively.

### Comparison with other zinc or MNP intervention trials on EED.

In the present study, we were unable to demonstrate any benefits of zinc supplementation on intestinal inflammation as assessed by fecal MPO, NEO, and CAL. Similarly, we did not find an impact of PZ and TZ supplementation or MNP on plasma concentrations of citrulline and the kynurenine: tryptophan (KT) ratio.^57^ Direct comparison of our findings with previous zinc or MNP intervention trials is made difficult by the use of different biomarkers of EED and different doses and combinations of supplementary micronutrients across the different studies. Two trials assessed the impact of MNPs with and without iron on fecal CAL among children in Kenya^40,41^; the first one found that fecal CAL was significantly higher in infants receiving iron-containing MNP (12.5 mg of iron as ferrous fumarate) than infants receiving MNP without iron, but no difference was reported with a MNP containing 2.5 mg of iron as NaFeEDTA compared with infants receiving the same MNP without iron.^40^ In the second trial, there was a nonsignificant decrease in fecal CAL in the MNP without iron group early (5 days) but not later (10–40 days) after supplementation compared with infants receiving iron-containing MNP (2.5 mg of iron as ferrous fumarate and 2.5 mg of iron as NaFeEDTA).^41^ Other trials assessed the L:M ratio, a marker of intestinal barrier permeability and absorptive capacity, and evidence from these studies is mixed.^35,38,39,58–60^ In a population of Bangladeshi children aged 3–6 months with acute and persistent diarrhea, 2-week zinc supplementation significantly reduced lactulose excretion, whereas the change in L:M ratio was similar in both zinc-supplemented and control groups,^58^ a finding replicated in a zinc supplementation trial in young rural Gambian children.^59^ In contrast, in 1–12 years old Bangladeshi children with a history of shigellosis, intestinal permeability as assessed by the L:M ratio improved significantly in vitamin B and zinc-supplemented children compared with that in children supplemented only with vitamin B syrup.^60^ However, among 12–35 months aged Malawian children, although there was an attenuation of the progression of EED with zinc or albendazole supplementation^35^ and a transient improvement of EED with MNP supplementation,^38^ a combined intervention of zinc, MNP, and albendazole did not ameliorate EED.^39^

### Reasons of absence of impact.

The overall lack of impact of zinc and MNP supplementation on these biomarkers of EED may be due to one of the following reasons: 1) the intervention had no impact on EED; 2) the duration of the intervention was not long enough, or adherence to supplementation protocol was too low; 3) the study participants were not sufficiently at risk of EED; and/or 4) the selected biomarkers are not sensitive makers of EED in this population. Each of these reasons will be addressed in detail in the following.

First, to the best of our knowledge, the present study is the first randomized controlled trial to assess the impact of PZ or TZ supplementation on intestinal inflammation as assessed by fecal MPO, NEO, and CAL, making any comparison with the existing literature difficult. We previously reported that although the provision of PZ increased zinc status and MNP increased both zinc and iron status,^50^ the interventions provided failed to improve linear growth,^50^ chronic stress as assessed by hair cortisol concentrations,^61^ and overall morbidity outcomes,^46^ although TZ supplementation did significantly reduce both the incidence and duration of diarrhea episodes in older children. However, this beneficial impact on diarrhea outcomes did not result in lower concentrations of MPO, NEO, and CAL in the TZ group nor did age modify the effect of the intervention on intestinal inflammation. In addition, there was no overall impact on other biomarkers of EED, namely, plasma citrulline and the KT ratio,^57^ suggesting that the intervention may have not affected intestinal damage and repair and systemic inflammation. Moreover, the modifying effects of EED on growth outcomes identified in the present study were no longer significant after multiple hypothesis testing, suggesting that these biomarkers of EED may not play a role in the pathway to linear growth failure in the present study population. Additional evidence is needed to corroborate this finding.

Second, the duration of the intervention could also explain the lack of impact observed in our study. Children participating in the present study were followed for ∼9 months, which is consistent with earlier studies that found a beneficial impact of zinc supplementation on functional zinc outcomes.^31,36,62^ However, this duration may have been inadequate to affect a complex outcome such as EED, especially intestinal inflammation, although the duration in previous zinc or MNP supplementation trials that reported an improvement of intestinal permeability ranged from as short as 2 weeks to up to 15 months.^35,38,39,58–60^ More evidence from supplementation trials is needed to understand whether zinc or MNP supplementation has a beneficial impact on intestinal inflammation as assessed by fecal MPO, NEO, and CAL. Poor adherence is unlikely the reason for our findings as reported adherence was high at > 90% and more importantly plasma zinc concentrations increased in both PZ and MNP groups, suggesting that the supplements were consumed and absorbed.^50^

A third reason for the lack of impact of our intervention may be that the study participants were not sufficiently at risk of EED to be able to respond to supplementation. There is currently no consensus about the MPO, NEO, and CAL cutoffs to be used to define EED. However, some studies have used the following standard values from either adults or children living in nontropical regions: MPO < 2,000 ng/mL, NEO < 70 nmol/L,^51^ and CAL < 100 µg/mL.^63^ The median baseline MPO, NEO, and CAL concentrations in our study were 2,710 ng/mL, 630 nmol/g, and 133 µg/mL, respectively, which correspond to 59%, 89%, and 59% of children having high values of MPO, NEO, and CAL using these previously used cutoffs. If these cutoffs are correct, this would suggest that most study participants had intestinal inflammation characteristic of EED, although these prevalences were lower than those of a recent study which reported high MPO and NEO in 71% and 97% of Bangladeshi children younger than 2 years^64^ and another study in Kenya in which 97% of children younger than 5 years had a high value of CAL.^65^ It is worth mentioning that zinc deficiency (75%) and stunting (39%) were very common in this study population.

Fourth, the selected biomarkers may not be sensitive indicators of EED in this population. No previous study has examined the impact of zinc supplementation on fecal MPO, NEO, or CAL. Previous zinc supplementation trials were inconsistent with some studies finding that supplementary zinc improved intestinal permeability as assessed by the L:M ratio,^35,60^ whereas others found no impact.^58,59,66^ Similarly, two previous studies investigated the impact of MNP on intestinal inflammation as assessed by CAL and found conflicting results,^40,41^ while MNP slightly improved the L:M ratio among Malawian children.^38^ It is possible that zinc or MNP affects specific aspects of the EED domain of intestinal permeability and absorption, but this does not translate into impacts on biomarkers of intestinal inflammation as measured by MPO, NEO, and CAL.

### Association between EED and subsequent or concurrent growth indicators.

According to the review by Harper et al.,^10^ there was strong evidence supporting the pathway between intestinal inflammation and linear growth in a variety of prospective and cross-sectional studies. In the present study, the finding that MPO concentrations at baseline were predictive of subsequent linear growth failure is consistent with some^22,67,68^ but not all previous studies.^69,70^ The inconsistency in these results may be due to the fact that in some of the aforementioned studies, EED biomarkers were combined to derive an EED disease activity score,^51^ or because growth indicators were assessed after different time periods. For example, we found an association between MPO and change in LAZ after 4.5 months but not after 9 months. In contrast, Kosek et al.^22^ found that children in the 75th percentile for MPO and NEO concentrations were predicted to have a decline in LAZ in the subsequent 6 months, whereas Arndt et al.^70^ reported that high fecal MPO levels in Bangladeshi children were associated with decreases in 3-month linear growth in the second year of life, and NEO levels were not associated with subsequent linear growth during any observed period in this analysis. Although fecal MPO, NEO, and CAL have shown promising results as biomarkers of EED, none of these biomarkers of intestinal inflammation was associated with concurrent LAZ or stunting in the present study. Only one previous cross-sectional study examined the association between CAL and stunting and found no association.^15^ In summary, the usefulness of fecal MPO, NEO, and CAL as potential screening tools for linear growth failure should be examined in other settings.

### Strengths and weaknesses.

A notable strength of this study includes its implementation in a setting with a high prevalence of zinc deficiency and stunting, and where EED is presumably endemic, and thus study participants should have had the potential to respond to the provided interventions. Another strength is its randomized placebo-controlled double-blind design, the frequency of follow-up visits, the high participation rate, and the rigorous data collection. This study is weakened by the fact that we were only able to assess intestinal inflammation. Unfortunately, because of logistical challenges, plasma citrulline, kynurenine, tryptophan, and the KT ratio were assessed in a different subsample of children,^57^ and we can thus not contribute to the comparison of different EED markers.

## CONCLUSION

Our data suggest that in this population of young Laotian children, the provision of supplementary zinc with or without other micronutrients had no overall impact on EED as assessed by fecal MPO, NEO, and CAL. In addition, these markers of intestinal inflammation appeared to have a minimal role in the pathways of growth faltering in this population. Additional research is needed to better understand the etiology and proposed mechanisms of EED pathogenesis.

## Supplemental tables and figures

Supplemental materials
