# Supplementary material for: Impact of Different Strategies for Delivering Supplemental Zinc on Selected Fecal Markers of Environmental Enteric Dysfunction among Young Laotian Children: A Randomized Controlled Trial
Source: Am J Trop Med Hyg. 2020 Jul 6;103(4):1416–26. doi: 10.4269/ajtmh.20-0106 (PMC7543857; doi:10.4269/ajtmh.20-0106)
Supplement: Supplementary file 1 [file tpmd200106.SD1.pdf]

Supplementary Tables

Supplementary Table 1: Associations between baseline MPO, NEO and CAL concentrations and concurrent growth indicators among young Laotian children

	**MPO[1]**		**NEO[1]**		**CAL[1]**	
	**Coefficient[2]**	**p-value**	**Coefficient[2]**	**p-value**	**Coefficient[2]**	**p-value**
Length	-0.01 (-0.04, 0.03)	0.734	0.00 (-0.04, 0.05)	0.824	0.02 (-0.03, 0.06)	0.424
Weight	-0.05 (-0.16, 0.06)	0.392	-0.01 (-0.13, 0.12)	0.918	0.04 (-0.09, 0.17)	0.566
MUAC	0.00 (-0.10, 0.11)	0.925	0.06 (-0.06, 0.18)	0.304	0.15 (0.02, 0.27)	**0.021**
Low MUAC (<=12.5 cm)	0.02 (-0.29, 0.34)	0.888	-0.12 (-0.48, 0.24)	0.506	-0.17 (-0.55, 0.21)	0.378
LAZ	-0.03 (-0.12, 0.07)	0.596	0.01 (-0.10, 0.12)	0.813	0.04 (-0.07, 0.16)	0.450
Stunting	0.07 (-0.13, 0.27)	0.512	0.05 (-0.18, 0.27)	0.678	-0.20 (-0.44, 0.04)	0.098
WAZ	-0.06 (-0.17, 0.04)	0.232	-0.01 (-0.13, 0.11)	0.886	0.02 (-0.10, 0.15)	0.723
Underweight	0.11 (-0.11, 0.33)	0.338	0.19 (-0.06, 0.44)	0.130	0.04 (-0.22, 0.30)	0.794
WLZ	-0.07 (-0.17, 0.03)	0.180	-0.04 (-0.16, 0.08)	0.531	-0.01 (-0.13, 0.12)	0.888
Wasting	0.18 (-0.16, 0.52)	0.296	-0.14 (-0.53, 0.24)	0.473	0.42 (0.02, 0.83)	**0.042**

[1]MPO, CAL and NEO concentrations are log-transformed.
[2]Estimates are regression coefficients and 95% confidence interval and models were adjusted for age at enrollment, sex and district of enrolment.

CAL: calprotectin; LAZ: length-for-age z-score; MPO: myeloperoxidase; MUAC: mid-upper arm circumference; NEO: neopterin; WAZ, weight-for-age z-score; WLZ, weight-for-length z-score.

Supplementary Table 2: Associations between endline MPO, NEO and CAL concentrations and concurrent growth indicators among young Laotian children

	**MPO[1]**		**NEO[1]**		**CAL[1]**	
	**Coefficient[2]**	**p-value**	**Coefficient[2]**	**p-value**	**Coefficient[2]**	**p-value**
Length	-0.01 (-0.03, 0.02)	0.465	-0.00 (-0.04, 0.04)	0.922	-0.03 (-0.07, 0.01)	0.197
Weight	-0.05 (-0.13, 0.02)	0.126	-0.00 (-0.11, 0.10)	0.897	-0.11 (-0.24, 0.00)	0.060
MUAC	-0.03 (-0.11, 0.04)	0.365	0.05 (-0.06, 0.17)	0.366	-0.10 (-0.24, 0.03)	0.130
Low MUAC (<=12.5 cm)	-0.17 (-0.49, 0.14)	0.279	0.00 (-0.48, 0.48)	0.996	-0.13 (-0.67, 0.41)	0.632
LAZ	-0.03 (-0.10, 0.05)	0.498	-0.00 (-0.12, 0.11)	0.890	-0.08 (-0.21, 0.05)	0.214
Stunting	0.08 (-0.05, 0.22)	0.232	0.01 (-0.20, 0.22)	0.898	0.16 (-0.07, 0.40)	0.177
WAZ	-0.06 (-0.14, 0.02)	0.143	-0.02 (-0.14, 0.10)	0.789	-0.14 (-0.28, -0.00)	**0.044**
Underweight	0.08 (-0.08, 0.25)	0.324	-0.06 (-0.31, 0.19)	0.626	0.32 (0.04, 0.60)	**0.024**
WLZ	-0.07 (-0.16, 0.01)	0.093	-0.02 (-0.15, 0.11)	0.806	-0.15 (-0.29, -0.00)	**0.044**
Wasting	0.00 (-0.34, 0.34)	0.984	0.37 (-0.15, 0.89)	0.162	0.14 (-0.44, 0.73)	0.629

[1]MPO, CAL and NEO concentrations are log-transformed.
[2]Estimates are regression coefficients and 95% confidence interval and models were adjusted for treatment group, age at endline, sex and district of enrolment.

CAL: calprotectin; LAZ: length-for-age z-score; MPO: myeloperoxidase; MUAC: mid-upper arm circumference; NEO: neopterin; WAZ, weight-for-age z-score; WLZ, weight-for-length z-score.

Supplementary Figures

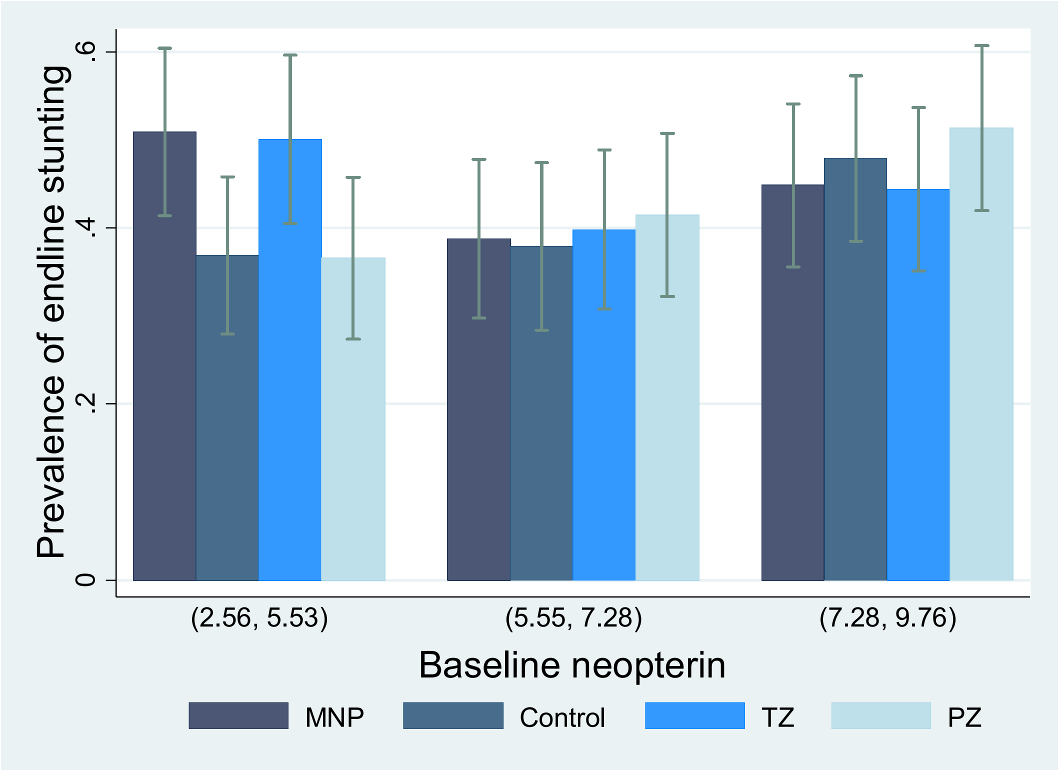

Supplementary Figure 1: Effect modification of baseline NEO concentrations on the impact of study intervention on endline stunting among young Laotian children. MNP=Micronutrient powder; PZ= preventive zinc; TZ=Therapeutic zinc.

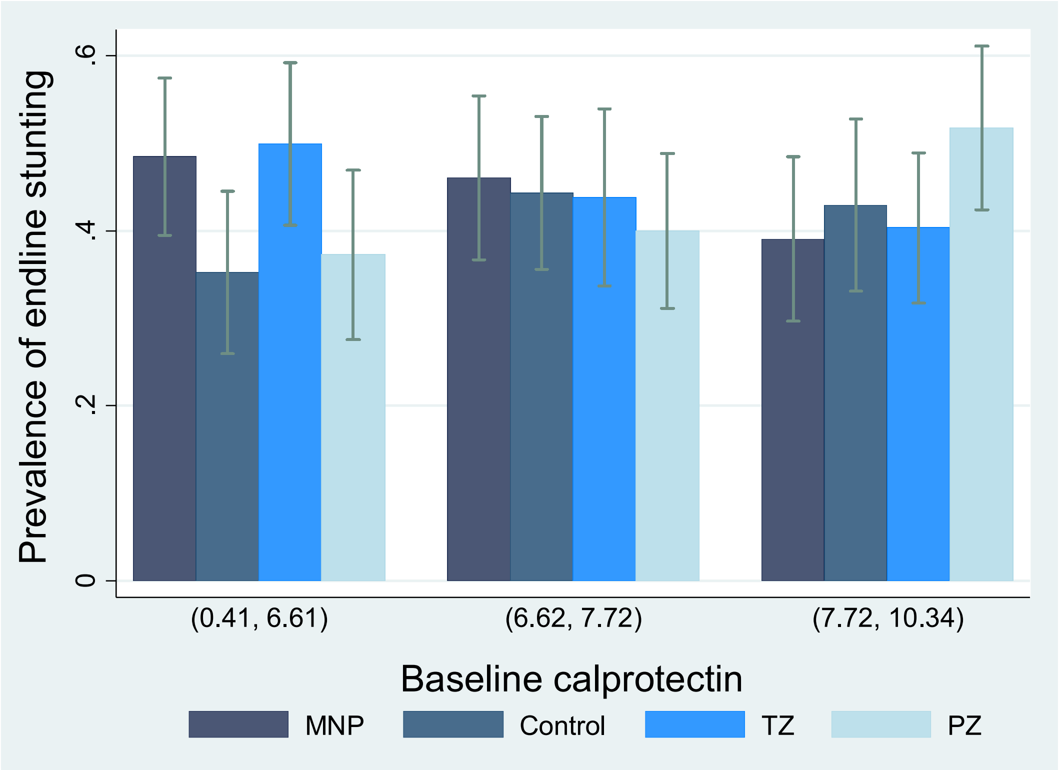

Supplementary Figure 2: Effect modification of baseline CAL concentrations on the impact of study intervention on endline stunting among young Laotian children. MNP=Micronutrient powder; PZ= preventive zinc; TZ=Therapeutic zinc.

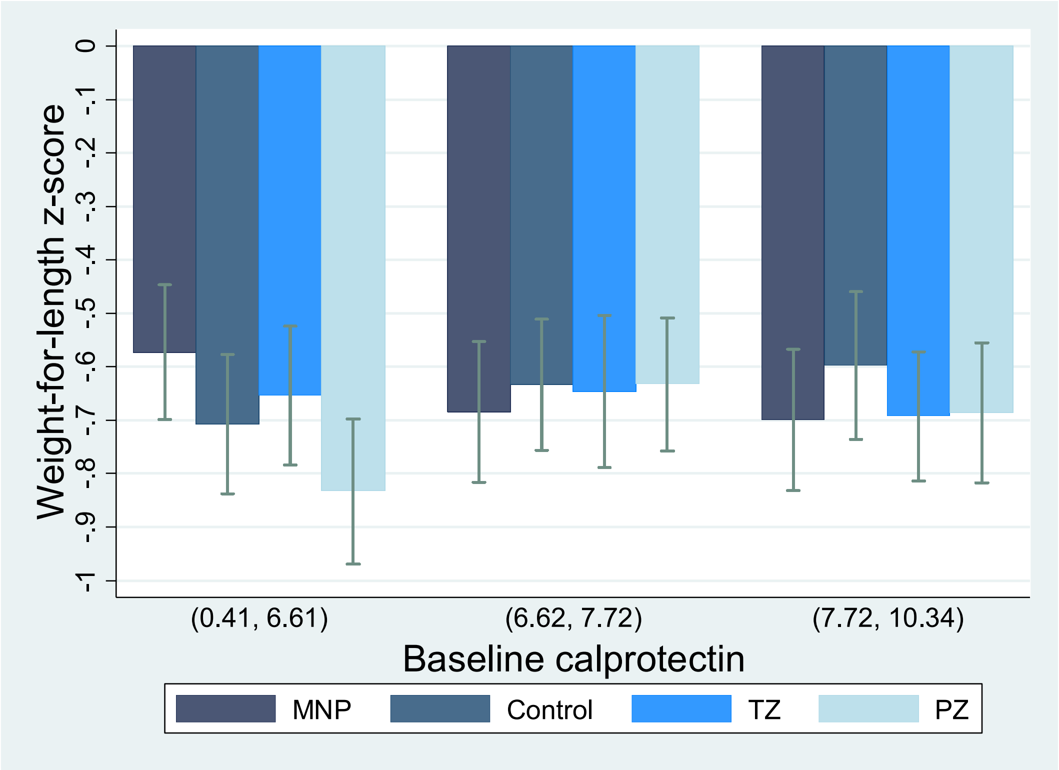

Supplementary Figure 3: Effect modification of baseline CAL concentrations on the impact of study intervention on endline weight-for-length z-score among young Laotian children. MNP=Micronutrient powder; PZ= preventive zinc; TZ=Therapeutic zinc
